# Modular megaprostheses in the treatment of periprosthetic fractures of the femur

**DOI:** 10.1007/s00508-021-01838-7

**Published:** 2021-04-13

**Authors:** Sebastian R. Apprich, Arastoo Nia, Markus M. Schreiner, Maximilian Jesch, Christoph Böhler, Reinhard Windhager

**Affiliations:** Department of Orthopaedic and Trauma Surgery, Medical University of Vienna, Waehringer Gürtel 18–20, 1090 Vienna, Austria

**Keywords:** Total knee arthroplasty, Total hip arthroplasty, Femur, Modular megaprosthesis, Limb salvage

## Abstract

**Background:**

Periprosthetic fractures (PPF) of the femur remain challenging, especially in patients with previous multiple revisions. Modular megaprostheses (mMPs) are rarely used in this indication; however, in some cases mMPs seem to be the last chance for limb salvage. We aimed to evaluate the clinical outcome of PPFs of the femur treated by modular mMPs at our institution.

**Patients and methods:**

In this study 33 patients (27 female; mean age 79 years) with a PPF after total hip or total knee arthroplasty (no tumor indications) were treated using modular proximal (mPFR; *n* = 12), distal (mDFR; *n* = 14) or total (mTFR; *n* = 7) femur replacement. A retrospective evaluation regarding mortality and revision rates was performed. Failures with need for revision were classified.

**Results:**

At a mean follow up of 60 months (range 0–178 months), the total mortality rate as well as total revision rate were both found to be 39%. At 1 year follow-up the mortality rate was highest within the mDFR group, and less revisions were necessary in the mPFR group, however both findings were not significantly. Those patients, who had revision surgery before PPF, were found to have higher revision rate after implantation of mMP. In the mPFR group, dislocation was the most frequent failure, within the mDFR and the mTFR group infection. In one case amputation of the lower limb was necessary.

**Conclusion:**

mMPs represent a valuable option in PPFs of the femur. Infection and dislocation remain the most frequent complications. Prospective clinical studies are required to further define the outcome of mMPs in PPFs of the femur.

## Introduction

Periprosthetic fractures (PPFs) of the femur are relatively rare events. The incidence after primary total hip arthroplasty (THA) is described as 0.1–1% and up to 20% after revision THA [1]. Similar findings are described around the knee joint with 0.3–2.5% after primary total knee arthroplasty (TKA) and 1.6–38% after revision TKA [2]; however, the continuing increase in the frequency of THA and TKA will result in a higher number of PPFs in the future [3, 4]. Therefore, a critical evaluation of potential treatment options for PPFs of the femur has to be made. While numerous studies deal with the surgical treatment of PPFs using open reduction and internal fixation with conventional non-locked plating, locked plating and retrograde intramedullary nailing, the reports of the use of modular megaprostheses (mMP) for endoprosthetic proximal (mPFR), distal (mDFR) or total femoral replacement (mTFR) are meager [5–7].

Evolution of so-called tumor or megaprostheses started in the late 1940s and the primary indication was the salvage of the limb in bone tumor cases. Since then, further developments were made with respect to the design as well as innovations for osteointegration, e.g. the introduction of the rotating hinge design substantially improved the revision rate for aseptic loosening. Later on modularity was introduced, which permits immediate intraoperative adaptation of the implant to the patients’ dimensions and produces wider ranges of implant options for reconstruction of segmental osseous defects.

Nowadays, megaprostheses are not restricted to tumor indications anymore. A wide expansion of indications for the use of megaprostheses in cases of fractures [8], massive bone loss, and aseptic or septic revision cases have been described [6]. In cases of PPFs of the femur, the endoprosthetic replacement by megaprostheses is an increasingly accepted salvage procedure, especially in older patients with loose implants and poor bone stock. Indication is mainly done by exclusion of other surgical modalities of joint reconstruction and taking into account the patient’s need for rapid recovery because of low activity levels and multiple comorbidities [7].

Recent studies described either a small number of patients or patients among a larger cohort who underwent implantation of a megaprostheses for different reasons than PPF. Therefore, the purpose of this study was to retrospectively evaluate the clinical outcome of patients who sustained a PPF of the femur and who were treated with a mMPs at our institution.

## Patients and methods

### Patient cohort

**Fig. 1:**
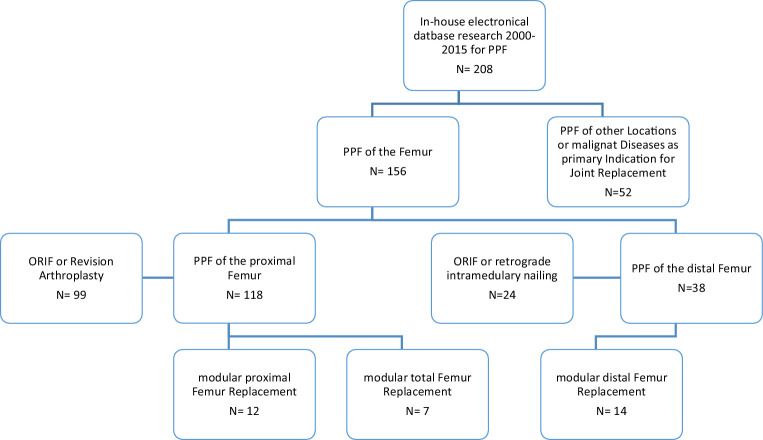
Flow chart of retrospective patient inclusion  – Open Reduction and Internal Fixation (ORIF), Periprosthetic fractures (PPF)

A digital search of the department’s electronic database and operation protocols was performed to identify all patients who were treated surgically in the course of a PPF of the femur at out institution between January 2000 and December 2015. From this initial pool of 208 patients, all PPF involving other bones than the femur as well as patients with malignant diseases as primary indication for joint replacement were excluded (*n* = 52). A total of 156 patients sustained a PPF of the femur either after THA (*n* = 118) or TKA (*n* = 38). Finally, after exclusion of all patients who were treated by either conventional ORIF (plating, cerclages, intramedullary nailing) or revision arthroplasty techniques, 33 patients were identified who received a mMP for treatment of PPF of the femur (mPFR *N* = 12; mTFR *N* = 7; mDFR *N* = 14). (Fig. 1).

Surgery was performed after each patient was medically optimized and informed consent was obtained. Due to the long retrospective follow-up period, patients were treated by variable surgeons; however, all were trained in arthroplasty and with experience in handling of MPs.

### Data assessment

Using our electronic medical record data base, the patient’s demographic details (age, gender, comorbidities), revision surgeries between primary implantation and the event of the PPF, surgical factors, hospital length of stay, postoperative rehabilitation protocol, postoperative complications and incidence of revision surgery were obtained retrospectively.

Failure of treatment with MP was defined as the need for surgical revision. The modes of failure were classified following the classification by Henderson [8]. The time point of the latest follow-up was defined from the date of the last documented visit in the outpatient clinic. The mortality rate was calculated with data from the registry of deaths from Statistics Austria (the Austrian federal institute for Statistics).

### Radiological assessment of PPF type

For classifying periprosthetic fractures near the hip joint, the Vancouver classification (types A–C) has been used, since it is currently the most accepted assessment scheme for periprosthetic proximal femoral fractures [9].

The standard classification system proposed by Su et al. is the one most commonly utilized for distal periprosthetic femoral fractures. This classification considers the fracture location in relation to the prosthesis [10].

### Statistical analysis

Statistical analysis was performed using the Statistical Package for Social Sciences, version 23.0 (SPSS Inc, Chicago, IL, USA) for Mac. Descriptive statistical evaluation was done by mean values, standard deviations, range values and percentage quotations. Kaplan Meier survival curves were used for visualization of either death or primary all-cause surgical revision as the endpoint. Differences in surgical revision rates and mortality were calculated using cross tables and χ^2^-tests for comparisons between groups of unranked categorical variables. *P*-values < 0.05 were seen as statistically significant.

## Results

### All patients

A total of 33 patients (27 females, mean age 79 years at time of megaprotheses implantation) were retrospectively enrolled in this study. In nearly all cases (*n* = 31) a low energy trauma was the reason for PPF of the femur, in 1 case a traffic accident and in the other case no reason was documented. The mean time between primary implantation of THA or TKA and first PPF was 84.9 months (Table 1).

**Table 1:** Patient demographics and radiological classification of Periprosthetic fractures (PPF) type

Patient number	Sex	Age at PPF (years)	Time between primary implant and PPF (months)	Radiological classification PPF	Comorbidities (*n*)
1	F	94	182	B3	1
2	F	74	Na	B3	4
3	F	80	5	B3	3
4	M	53	105	B3	9
5	F	85	34	B3	7
6	M	88	2	C	5
7	F	79	1	B3	2
8	F	87	155	B3	1
9	F	78	68	B2	1
10	F	77	117	B3	7
11	F	80	2	B3	4
12	F	77	105	B2	6
13	F	77	164	3	3
14	M	86	43	3	4
15	F	70	28	3	5
16	F	72	64	3	7
17	F	81	Na	3	6
18	F	60	0	Na	4
19	F	81	83	Na	4
20	F	83	69	3	5
21	F	65	29	3	4
22	F	82	62	Na	5
23	F	89	197	3	5
24	F	95	218	3	6
25	F	86	179	3	5
26	F	87	Na	2	6
27	M	84	316	B3	9
28	F	73	2	Na	3
29	F	81	72	B3	1
30	M	60	40	Na	5
31	F	67	80	B3	1
32	F	76	44	C	6
33	M	68	80	B3	3

At a mean follow up of 60 months (range 0–178 months) total mortality rate was 39.4%.

**Fig. 2:**
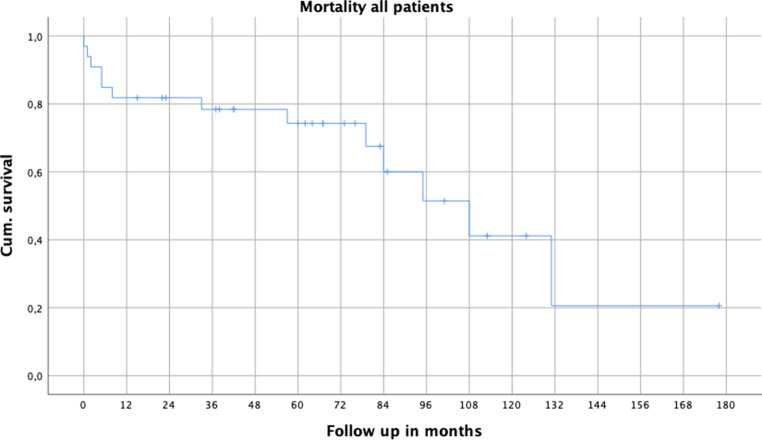
Kaplan Meier cumulative (cum) survival curve for all patients with death as endpoint

The 1‑year mortality was 18.2%, and after 3 years 21.2% of patients had died (Fig. 2).

**Fig. 3:**
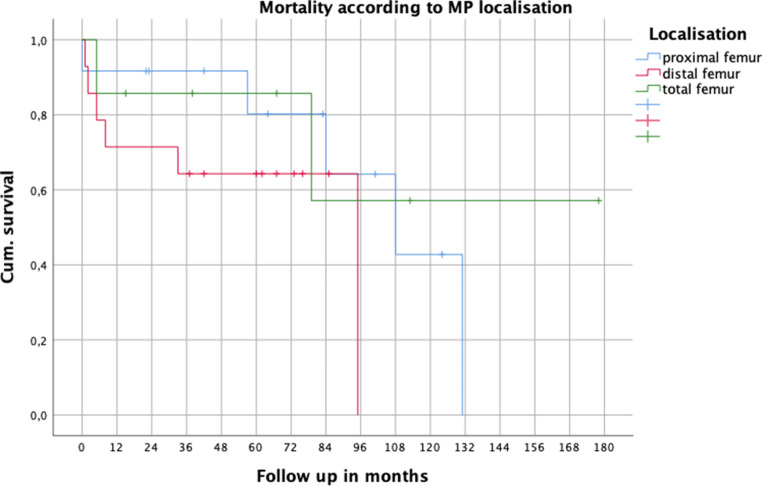
Mortality according to MP location

After stratification of treatment to mPFR, mDFR and mTFR, we found a higher mortality rate for the patients with distal femur after 1 year compared to proximal and total femur; however, without statistical significance (*p* = 0.326) (Fig. 3).

**Fig. 4:**
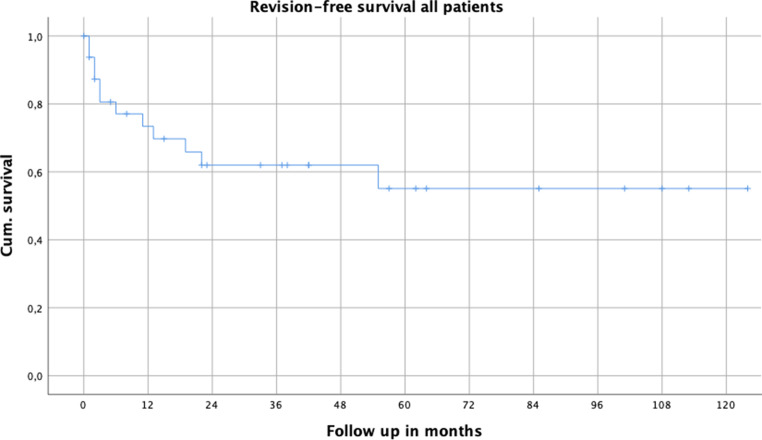
Kaplan Meier survival curve for all patients with revision surgery as endpoint

**Fig. 5:**
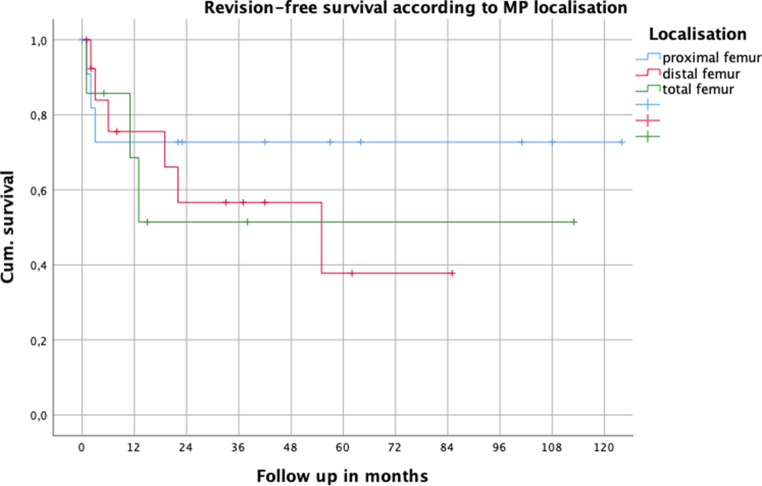
Revision-free survival according to MP location

In 12 patients (36.4%) 1 or more revision surgeries (range 1–5) were necessary, with 26 surgical revisions in total. Revision-free survival was 73% after 1 year, and 62% after 3 years (Fig. 4). After 1 year mPFR seemed to have a better revision free outcome compared to mDFR and mTFR; however, again not statistically significant (Fig. 5). In those patients without surgical revision between initial implantation of THA or TKA and PPF, the surgical revision rate after MP treatment was significantly lower (22.7%; 5 out of 22) than in patients with surgical revision between implantation and PPF (63.6%; 7 out of 11) (χ^2^-test*p* = 0.021; moderate correlation Cramer V 0.401, *p* = 0.021). Furthermore, 26.3% of patients who were treated with MP for PPF as a first line treatment needed surgical revision after PPF, whereas in cases of secondary treatment by MP 50% of the patients were surgically revised. According to χ^2^-test this finding was not significant (*p* = 0.162).

No difference was found concerning the mortality rate, either for the influence of surgical revision between initial implantation of THA or TKA and PPF (*p* = 0.614) or treatment with MP as first or second line treatment (*p* = 0.284).

### Detailed results for mPFR

**Fig. 6:**
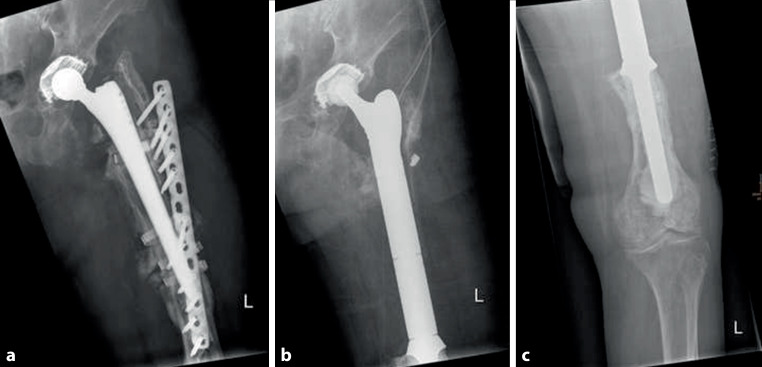
An 87-year-old female patient with multiple revision surgeries between primary implantation of THA and PPF. The initial PPF was treated by cerclages and a lateral locking plate **a** which failed after 9 months. Proximal femur reconstruction included implantation of cemented proximal femur GMRS protheses **b, c** with preservation of proximal trochanter structures using one super cable and multiple transosseus fiber wires and change of femoral head

Of the patients 12 (10 female; mean age at time of PPF 79.3 years, range 53–94 years) were treated by mPFR (6 × Stryker [Orthopaedics, Mahwah, NJ, USA] proximal Femur GMRS, 6 × proximal Femur KMFTR), (Fig. 6).

In 9 patients PPF was classified as Vancouver type B3 (1 type B2, 1 type C, 1 not classified). 3 patients needed revision of primary implant before PPF. In 6 patients PPF was initially treated by mPFR, whereas in 6 patients PPF was first treated by ORIF. Reason for failure in primary ORIF cases was additional PPF in 3 cases, plate breakage caused by nonunion in 2 cases and septic revision in one case. Mean time between first treatment of PPF by ORIF and implantation of megaprosthesis was 13.2 months (range 1–45 months).

The mean postoperative inpatient stay after mPFR was 21 days (range 7–45 days), and postoperative mobilization was performed with full weight bearing in 10 patients.

The 1‑year and 3‑year mortality rates for patients after mPFR was 8.3%, and total mortality rate at a mean follow up of 69.9 months was 41.7%.

Three patients (25%) needed revision surgery (total *n* = 5), in all cases due to hip dislocation (type 1 failure). All patients were treated with open reposition and one patient received an additional change of inlay and plaster cast afterwards. At the time of follow-up, limb salvage was possible in all cases. (Table 2).

**Table 2:** Clinical data of patients treated with mPFR

Pat. Nr.	Number revision surgeries between primary Implant and PPF	Initial treatment PPF	Number of revision surgeries after PPF and before MP	Indication for MP	Implant type MP	N Revision surgeries after MP	Ambulation at Discharge	Failure mode	FU (months)
1	1	MP	–	PPF	pfKMFTR	–	Partial weight bearing	–	42
2	–	MP	–	PPF	pfKMFTR	–	Full weight bearing	–	101
3	–	MP	–	PPF	pfGMRS	–	Full weight bearing	–	108(†)
4	1	MP	–	PPF	pfGMRS	2	Full weight bearing	Type 1	131(†)
5	–	ORIF	–	Second PPF	pfKMFTR	–	Full weight bearing	–	23
6	–	ORIF	1	Nonunion after ORIF	pfGMRS	–	Partial weight bearing	–	57(†)
7	–	MP	–	PPF	pfKMFTR	–	Full weight bearing	–	64
8	2	ORIF	1	Nonunion after ORIF	pfGMRS	1	Full weight bearing	Type 1	84(†)
9	–	ORIF	–	Failed ORIF	pfKMFTR	–	Full weight bearing	–	22
10	–	ORIF	–	Failed ORIF	pfKMFTR	–	Full weight bearing	–	0(†)
11	–	MP	–	PPF	pfGMRS	–	Full weight bearing	–	124
12	–	ORIF	3	Replantion	pfGMRS	2	Full weight bearing	Type 1	83

*†* Patient diseased, *MP* Megaprosthesis, *PPF* Periprosthetic fractures, *ORIF* Open Reduction and Internal Fixation, *pfGMRS* proximal femur Global Modular Replacement System, *pfKMFTR* proximal femur Kotz Modular Femur Tibia Reconstruction

### Detailed results for mDFR

**Fig. 7:**
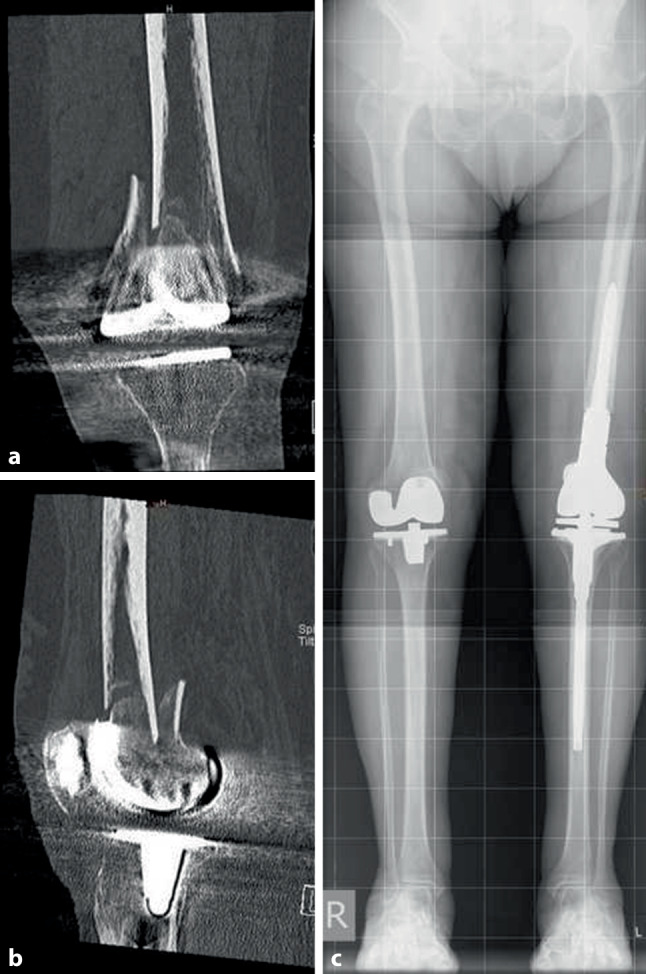
A 77-year-old female patient with PPF of the distal femur. CT scans verify type 3 fracture according to Su et al. Treatment with modular distal femur GMRS allowed for reconstruction of correct leg length and joint line

Of the patients 14 (13 female; mean age at time of PPF 78.5 years, range 60–95 years) were treated by mDFR (Stryker distal Femur GMRS). (Fig. 7) In 10 patients PPF was classified as type 3 after Su et al. (1 type 2, 3 not classified) [11] and 5 patients needed revision of primary implant before PPF (3 × aseptic loosening, 2 × septic revision).

In 10 patients PPF was directly treated by mDFR, whereas in 4 patients PPF was primarily treated by ORIF. Reason for failure in primary ORIF cases was plate breakage, septic revision, repeated PPF and nonunion, respectively. Mean time between first treatment of PPF by ORIF und implantation of megaprosthesis was 5.5 months (range 1–13 months). The mean postoperative stay at hospital was 23 days (range 8–59 days), and postoperative mobilization was performed with either full or partial weight bearing in 7 patients. The 1‑year and 3‑year mortality rates for patients with mDFR were 26.7% and 33.3%, respectively, total mortality rate at a mean follow-up of 46.1 months was 42.9%, 6 patients (42.9%) needed revision surgery, in 3 cases due to infection (type 4 failure), in 2 cases due to aseptic loosening (type 2) and in 1 case due to further PPF (type 3). In one patient, an exarticulation of the hip had to be performed due to an imminent sepsis. In total 15 revision surgeries had to be performed within the mDFR group. (Table 3).

**Table 3:** Clinical data of patients treated with mDFR

Pat. Nr	Number of revision surgeries between primary Implant and PPF	Initial treatment PPF	Number of revision surgeries after PPF and before MP	Indication for MP	Implant type MP	N revision surgeries after MP	Ambulation at Discharge	Failure mode	FU (months)
13	–	MP	–	PPF	dfGMRS	–	Partial weight bearing	–	62
14	2	MP	2	PPF	dfGMRS	–	Partial weight bearing	–	8 (†)
15	–	MP	–	PPF	dfGMRS	2	Full weight bearing	Type 4	76
16	–	ORIF	–	PPF	dfGMRS	3	Partial weight bearing	Type 4	5 (†)
17	1	MP	1	PPF	dfGMRS	–	Full weight bearing	–	85
18	2	ORIF	2	Septic Revision	dfGMRS	5	Full weight bearing	Type 4	73
19	–	IM	–	Non union	dfGMRS	1	Full weight bearing	Type 2	95 (†)
20	1	MP	1	PPF	dfGMRS	1	Partial weight bearing	Type 2	67
21	1	MP	1	PPF	dfGMRS	1	Partial weight bearing	Type 3	60
22	–	ORIF	–	Failed ORIF	dfGMRS	–	Full weight bearing	–	33 (†)
23	–	MP	–	PPF	dfGMRS	–	Partial weight bearing	–	37
24	–	MP	–	PPF	dfGMRS	–	Full weight bearing	–	2 (†)
25	–	MP	–	PPF	dfGMRS	–	Full weight bearing	–	42
26	–	MP	–	PPF	dfGMRS	–	Partial weight bearing	–	1 (†)

*MP* Megaprosthesis, *PPF* Periprosthetic fractures, *ORIF* Open Reduction and Internal Fixation, *dfGMRS* Distal Femur Global Modular Replacement System

### Detailed results for mTFR

**Fig. 8:**
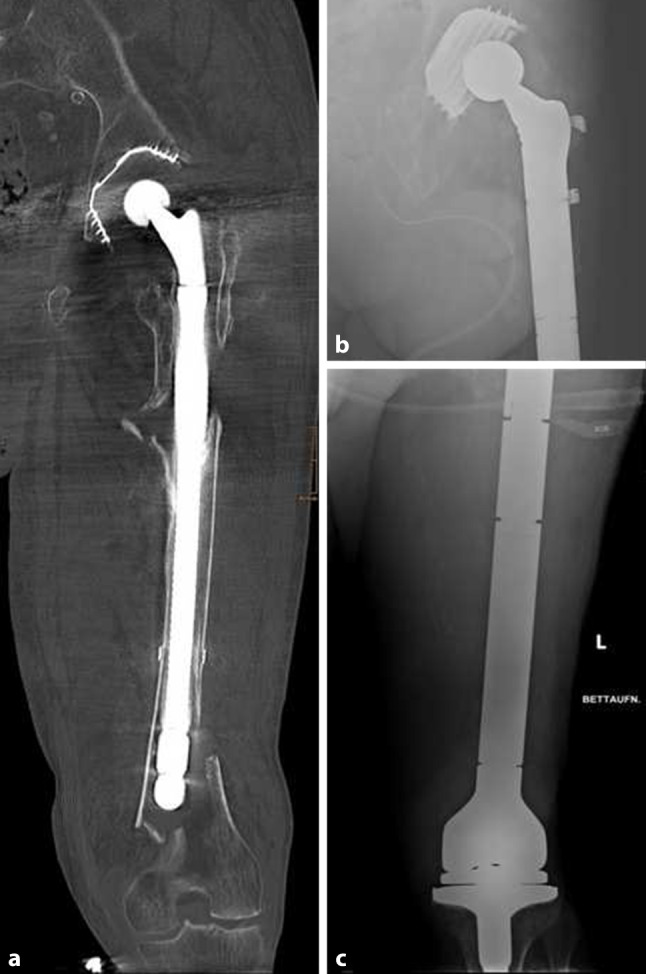
An 84-year-old male patient with multiple revision surgeries before suffered a type B3 fracture according to the Vancouver classification. mTFR was performed by total femur GMRS and attachment of the remaining structures of the trochanter major to the prothesis by 2 supercables and fibre wires

Of the patients 7 (4 female; mean age at time of PPF 73 years, range 60–84) were treated by mTFR (3 × Stryker total Femur GMRS, 3 × Stryker total Femur KMFTR, 1 × Stanmore total Femur). (Fig. 8). In 4 patients PPF was classified as Vancouver type B3 (1 type C, 2 not classified). In all patients, initial treatment before PPF involved the hip joint (4 × THA, 1 × proximal femur GMRS due to infection and 2 × dynamic hip screw) and all patients had multiple revision surgeries (total 25; mean 3.6; range 2–7) between initial treatment and mTFR.

In 3 patients PPF was initially treated by mTFR, whereas in 2 patients PPF was first treated by ORIF and in 2 patients by long stem hip protheses. Reasons for failure which led to secondary mTFA were plate breakage, septic revision, further PPF and nonunion. Mean time between first treatment of PPF by ORIF und implantation of megaprosthesis was 5.5 months (range 1–13 months).

The mean postoperative stay of these patients at hospital was 35 days (range 10–73 days), and postoperative mobilization was performed with full weight bearing in 4 and partial weight bearing in 3 patients. The 1‑year and 3‑year mortality rates for patients with mTFR was 14.3%, total mortality rate at a mean follow-up of 70.7 months was 28.6%.

Three patients (42.9%) needed revision surgery (in total *n* = 10) after mTFR. In 2 cases due to infection (type 4) and in one case due to dislocation in the hip joint (type 1). At the time of follow-up, limb salvage was possible in all cases (Table 4).

**Table 4:** Clinical data of patients treated with mTFR

Pat. Nr	Number of Revision Surgeries between primary Implant and PPF	Initial Treatment PPF	Number of Revision Surgeries after PPF and before MP	Indication for MP	Implant Type MP	Number of Revision Surgeries after MP	Ambulation at Discharge	Failure Mode	FU Months
27	3	MP	–	PPF	tfGMRS	–	Partial weight bearing	–	5 (†)
28	–	ORIF	3	Septic Revision	Silver coated tfStanmore prosthesis	–	Full weight bearing	–	38
29	4	ReAP	4	Septic Revision + PPF	tfGMRS	–	Full weight bearing	–	15
30	4	ORIF	4	PPF	tfKMFTR	5	Full weight bearing	Type 1	79 (†)
31	1	ORIF	3	Septic Revision+ PPF	tfKMFTR	3	Partial weight bearing	Type 4	67
32	3	MP	3	PPF	tfGMRS	–	Full weight bearing	–	113
33	7	MP	7	PPF	tfKMFTR	2	Partial weight bearing	Type 4	178

*MP* Megaprosthesis, *PPF* Periprosthetic fractures, *ORIF* Open Reduction and Internal Fixation, *tfGMRS* Total Femur Global Modular Replacement System, *tfKMFTR* Total Femur Kotz Modular Femur Tibia Reconstruction

## Discussion

The indication for endoprosthetic replacement by a mMP in case of PPF of the femur is mostly given when other surgical options are not feasible. Eligible patients typically present with loose prosthesis components, poor metaphyseal bone stock, and advanced age (low demand). This implicates a need for rapid recovery from bed with opportunity of full weight bearing. The therapeutic goal for these patients is to return them to their preinjury ambulation status. It is considered as a limb salvage procedure despite the known high mortality and revision rates.

In the present study, the 1‑year mortality rate for all patients was 18.2%. This is comparable to the mortality rates described in other studies for treatment of PPF of the femur with conventional osteosynthesis and arthroplasty techniques [11]. These high mortality rates originate, beside the complications which occur with the treatment of megaprotheses, from the fact that these patients most often suffer from multiple comorbidities. Advanced age has already been identified as an independent risk factor of mortality before [12]. A continuous preoperative and postoperative preparation and internal care of these patients as well as rapid postoperative ambulation might be a possibility to reduce the high 1‑year mortality rate.

Regarding the high number of revision surgeries, the surgical challenge in this context is to minimize possible necessary follow-up surgeries. Therefore, a critical evaluation of subsequent failure modes is indispensable. In the present study, dislocation of the hip joint was the leading cause for revision surgery after mPFR. This is consistent with the current literature. A systematic literature review by Korim et al. [13] summarized 14 studies, which described the clinical results of proximal femur replacement in nonneoplastic indications. Out of 356 patients, 96 patients suffered from a PPF of the proximal femur. The surgical revision rate in this patient group ranged from 13.3% to 40%. Leading cause of failure in all patients was hip dislocation (15.7%), followed by infection (7.6%). A more recent study from Visite et al. dealing with proximal femoral replacement in contemporary revision total hip arthroplasty for severe femoral bone loss (34% PPFs), confirmed this finding with a 14% failure rate due to dislocation in all patients [14]. In contrast, a recent study by Grammatopoulos et al. describing the clinical results of proximal femoral endoprosthetic arthroplasty for nontumor indications, found periprosthetic joint infection to be the predominant complication; however, in this cohort nearly 50% of patients showed a periprosthetic joint infection before, and only 15% were patients suffering from PPF [15]. Colman et al. compared the mortality and implant survivorship of different treatment options (PFR vs. revision total hip arthroplasty vs. ORIF) for acute PPF of the proximal femur [16]. Whereas the mortality during a 35-month follow-up showed no difference between the three groups, the implant survival and reoperation rate was worse for the PFR group, primarily due to instability and dislocation. The authors concluded that the enthusiasm for expanded use of PFR in cases of PPF of the proximal femur should be tempered, although they state that in certain situations PFR may be the only reasonable reconstruction modality.

A different challenge poses the results from this and former studies concerning the failure modes after mDFR. Here, the predominant complication seems to be the periprosthetic joint infection (PJI). In a review of Windhager et al. [17] including 8 studies, the total surgical revision rate for all patients who were treated by DFR for PPF of the distal femur, was found to be 31%. Nearly 50% of these were caused by PJI, followed by 29% due to type 3 failure (mostly further PPF). In case of reasonable suspicion for a PJI, preoperative biopsy and identification of an infecting micro-organism are essential as well as a radical debridement at the time of surgery.

Better outcomes were described for patients, who are treated in case of PPF of the distal femur by DFR in the first course than in case of failed ORIF. In this context, a recent study on a comparatively large number of patients compared the results of periprosthetic distal femoral fractures initially treated by either locked lateral plating (LLP) or DFR. The authors found that the 90-day and 365-day mortality, final mobility, and reoperation rate were not statistically different with LLP vs DFR management [13]; however, interestingly patients in the LLP group, who survived 1 year were significantly younger than those who died (77 vs. 85 years, *p* < 0.01). This difference was not found for DFR patients.

Patients who fail further standard fracture care may eventually progress to distal femoral replacement (DFR), and these patients continue to have a higher rate of complications than patients initially managed with DFR, have incurred a greater cost, and have endured multiple major surgeries [12]. Prospective studies might clarify if patients with peri-TKA femoral fractures (especially in type 3 fractures after Su et al.) benefit from index DFR management instead of LLP?

Concerning mTFR for PPF, similar results to our study were described before. Besides the likewise high number of dislocations, infection is also a second leading cause for revision surgery in this group of patients. This is explained by the fact that most patients who were treated by a TFR already had multiple revision surgeries before.

The largest study with 20 patients treated with a TFR after periprosthetic fracture was published by Clement et al. [19]. The indication in most cases was due to pseudarthrosis after failed ORIF. The 10-year mortality was 58% and the 10-year implant survival was 86%.

Toepfer et al. [18] collated the outcome after TFR due to 11 periprosthetic femoral fractures and 7 aseptic loosening of the prosthesis in a mean follow-up of 80 months. All patients had undergone multiple operations before TFR. There was a rate of 44% implant failure after 5 years. In total, only 5 patients remained revision-free, resulting in a complication rate of 72%. The majority of the revision operations had to be performed due to dislocations. Failure due to infection was the second most common reason. The authors concluded that the high complication and revision rates after total femur replacement require strict indication and careful consideration of the advantages and disadvantages of this treatment option.

Amanatullah et al. reported about 20 patients in their study who were treated with TFR. In 35% of the cases (7/20) the indication was due to a PPF, in 50% of the cases due to an existing infection (10/20). Again, the two primary reasons for failure were infection and instability [20].

Our study is limited by its retrospective nature and relatively small number of patients. The relatively low incidence of PPF of the femur, especially when treatment is performed by mMP, makes this a difficult patient cohort to study in large numbers. Furthermore, we cannot provide comprehensive postoperative functional scoring of our patients.

In conclusion, reconstruction and salvage procedures by megaprostheses following periprosthetic fractures of the femur could be a reliable and effective solution and allow for early weight bearing mobilization and return to activities of daily living; however, infections and dislocation remain the most dominant complications after for nonneoplastic indications such as the PPF. Therefore, it should be considered as an available solution in extreme and appropriately selected cases only. It is important that careful patient selection is applied and that accurate preoperative planning is utilized to minimize revision rate. This type of complex surgery should be performed in specialized centers only where sufficient financing, knowledge and technologies are present.

However, prospective clinical studies are required to exactly define the outcome of mMP in PPFs of the femur.
